# A deep learning model for the localization and extraction of brain tumors from MR images using YOLOv7 and grab cut algorithm

**DOI:** 10.3389/fonc.2024.1347363

**Published:** 2024-04-12

**Authors:** Srigiri Krishnapriya, Yepuganti Karuna

**Affiliations:** School of Electronics Engineering, Vellore Institute of Technology, Vellore, India

**Keywords:** brain tumor, deep learning, YOLOv7, grab cut algorithm, magnetic resonance imaging (MRI), gamma correction

## Abstract

**Introduction:**

Brain tumors are a common disease that affects millions of people worldwide. Considering the severity of brain tumors (BT), it is important to diagnose the disease in its early stages. With advancements in the diagnostic process, Magnetic Resonance Imaging (MRI) has been extensively used in disease detection. However, the accurate identification of BT is a complex task, and conventional techniques are not sufficiently robust to localize and extract tumors in MRI images. Therefore, in this study, we used a deep learning model combined with a segmentation algorithm to localize and extract tumors from MR images.

**Method:**

This paper presents a Deep Learning (DL)-based You Look Only Once (YOLOv7) model in combination with the Grab Cut algorithm to extract the foreground of the tumor image to enhance the detection process. YOLOv7 is used to localize the tumor region, and the Grab Cut algorithm is used to extract the tumor from the localized region.

**Results:**

The performance of the YOLOv7 model with and without the Grab Cut algorithm is evaluated. The results show that the proposed approach outperforms other techniques, such as hybrid CNN-SVM, YOLOv5, and YOLOv6, in terms of accuracy, precision, recall, specificity, and F1 score.

**Discussion:**

Our results show that the proposed technique achieves a high dice score between tumor-extracted images and ground truth images. The findings show that the performance of the YOLOv7 model is improved by the inclusion of the Grab Cut algorithm compared to the performance of the model without the algorithm.

## 1 Introduction

Brain tumors (BT) result in an unusual growth of brain cells, which is caused by the uncontrolled division of cells in the brain. In general, BTs are categorized as malignant (cancerous) or benign (noncancerous). (1). Benign or normal tumors do not cause any damage to the brain cells and can be easily treated. On the other hand, malignant tumors are dangerous and can spread to other organs if not treated in the early stages. The tumors are also classified as primary and secondary tumors wherein primary BT is developed from the existing cells and secondary tumors are developed from the cancerous cells (2). Benign tumors develop slowly and can be identified easily. These tumors can be removed by determining the brain region where they are located. Conversely, brain tumors can have serious consequences on human health and do not have any specific boundaries. Hence, they can affect other healthy cells in the brain and thereby completely disrupt the functioning of the brain (3).

There are several imaging modalities such as Perfusion magnetic resonance imaging (4), computed tomography (CT) (5), and positron emission tomography (PET) (6). Among the different techniques, MRI is a potential technique for identifying irregularities in brain patterns and works effectively on soft tissue (7). MRI is an invasive technique that generates high-quality brain images with better resolution. Usually, brain tumors are treated using advanced treatment processes such as chemotherapy, radiotherapy, and surgery which can destroy cancerous cells completely if the location of the tumor is identified correctly (8–10).

Manual identification and diagnosis of brain tumors can be a tedious and labor-intensive task. Since these techniques depend on manual intervention, the accuracy and precision of the tumor detection process are questionable. Hence, there is a need for a qualitative approach that can detect tumors and their location in the early stage with high accuracy and precision (11–13). The use of machine learning (ML) and deep learning (DL) techniques for precisely detecting BT has been emphasized in several studies (14, 15).

ML algorithms such as support vector machines (SVM) (16), Random Forest (RF) (17), Decision Trees (DT), K-nearest neighbor (KNN) (18), etc. have been used in previous works. However, these algorithms depend on manual feature extraction wherein the detection models are trained using these features. Hence, the accuracy of the detection and classification of brain tumors depends on the quality of the extracted features. In addition, constructing ML classifiers requires more resources, and their computational time is very high while processing large-scale datasets. As a result, these models exhibit a low classification accuracy (19).

A substantial amount of research has been dedicated to brain tumor detection and segmentation processes and various researchers have attempted to address the complexities associated with the detection process (20–22). One of the main challenges related to brain tumor identification is the classification of neoplastic tissues which are heterogeneous in nature. These tissues overlap with the healthier tissues most of the time and conventional techniques used for tumor detection fail to distinguish them. Texture analysis is one such effective technique that can be used to determine the textural features of the tumors such as regularity, and orientation of the tumor, and thereby identify multiple indistinct areas in an image (23, 24).

The extraction of textural features helps the classifier to determine both visible and non-visible tumor regions with the aid of advanced techniques such as MRI. Conventional ML classifiers use gray-level and pixel-level-based features for classifying malignant and benign tumors. Various algorithms are used to automatically segment BT using MRI images and these techniques fail to achieve desired solutions for the issues related to BT detection techniques (25). The hybrid Convolutional Neural Network (CNN) and Deep Neural Network (DNN) were suggested (26) for addressing the drawbacks of ML algorithms such as high computational time and reduced classification accuracy. In this process, the CNN model was used to extract features that were classified using a fully connected network. The DNN employed in this work enhanced the performance of CNN by accurately classifying the tumor regions with an accuracy and F1 score of 96.08% and 97.3% respectively.

An ensemble model is implemented for distinguishing BT from MRI images (27). A pre-trained Inception ResNetV2 model is adopted for tumor detection and a ML-based RF model is employed for determining the stage and type of brain cancer (28). A cycle generative adversarial networks (C-GAN) model is used to augment the size of the dataset. The results exemplify that the proposed ensemble approach achieved detection and classification accuracies of 99% and 98%, respectively. The authors Dipu et al. (29) implemented a YOLOv5 model for detecting BT along with a DL library known as FastAi. The model was trained using data collected from the BRATS 2018 dataset, which consisted of 1,992 brain MRI images. It attained an overall accuracy of 85.95% and the FastAi model exhibited an improved accuracy of 95.78%. These two techniques validated the effectiveness of DL in the early detection of brain cancer.

The work mentioned in (30) implemented a YOLOv3 for identifying cancerous BTs. The YOLOv3 model was combined with a CNN model to boost the performance. This hybrid model attained an accuracy of 97%. However, YOLOv3 significantly requires more memory and this can be a challenging factor while working with limited resources. A YOLOv4 model is employed in (31) for BT detection. It is trained using a transfer learning (TL) approach and a pre-trained COCO dataset was used to maximize the tumor detection performance. Compared to the traditional YOLO model, the YOLOv4 model achieves better performance but with a high localization error.

A YOLOv5 was used by Paul et al. and Shelatkar et al. (32, 33) for segmenting brain cancer images and diagnosing brain tumors. The YOLOv5 was trained on the BRATS 2021 dataset and the model achieved an average precision of 88%. It was observed from the results that the YOLOv5 model provided a slightly lesser accuracy compared to other classification models. It was also inferred that the complexity of the model increases the training time. The authors Arunachalam & Sethumathavan and Hossain et al. (34, 35) implemented YOLOv5 to detect abnormalities from brain images. The YOLOv5 model performed better compared to previous versions of YOLO and exhibited excellent tumor detection performance. However, the model was not tested for detecting malignant tumors from brain images.

As inferred from the existing approaches, most of the techniques used in the brain tumor detection process employ pre-processing and segmentation to identify and distinguish BTs and these techniques are not effective in recognizing normal or malicious tumor areas. In addition, conventional YOLO models namely YOLOv3, YOLOv4, and YOLOv5 suffer from certain drawbacks such as high computational complexity, compromised accuracy to maintain fast execution speed restricts their adaptability in disease detection tasks. Besides, these models rely on larger datasets, and collecting such datasets for rare tumor classes can be a tedious and time-consuming task. These drawbacks motivate this research to employ an advanced version of the YOLO model to automatically segment BTs with enhanced accuracy.

To address these drawbacks, in this work, we implemented a deep learning (DL) model for the accurate detection of brain tumors with better performance. The detection and segmentation of brain tumors from MRI images using the hybrid DL-based YOLOv7 and Grab Cut algorithms are presented here. The model was trained using a Br35H Brain tumor dataset, and its effectiveness is validated through a comparative analysis.

The primary contributions of the proposed work are as follows:

- The data processing technique used in this work consists of different processes such as RGB to Gray conversion, Otsu’s thresholding, Brain Skull Removal, Image Resizing, and Median filtering.
- We proposed an efficient object detection-based YOLOv7 algorithm for diagnosing brain tumors in the early stages to mitigate the effect and speed up the diagnosis process.
- A gamma correction technique and a Grab Cut algorithm are used to extract the Gamma-corrected image.
- The performance of the YOLOv7 model is evaluated with and without the GrabCut algorithm and the proposed model performed better than the other existing algorithms in both cases.

The remaining portion of the paper is organized as: Section 2 includes the suggested methodology to train the model with the sourced dataset for detecting tumors from brain MRI images. This section also discusses the implementation of YOLOV7 and the GrabCut algorithm for the detection and extraction of tumors. Section 3 evaluates the results of the experiments conducted based on the proposed methodology. Lastly, Section 4 outlines the conclusion based on the produced results with future scope.

## 2 Materials and methods

This paper aims to achieve a highly accurate recognition of BTs from MRI images. DL-based YOLOv7 model (36) is used for achieving faster and more accurate results for tumor detection and classification. The automatic recognition of BT is a challenging task because of the similarities and irregularities in tumor images obtained from MRI scans. These issues make it difficult for the classifier to recognize and classify the tumors with better precision. Hence, it employs a Gamma correction mechanism to improve the quality of the images.

**Figure 1:**
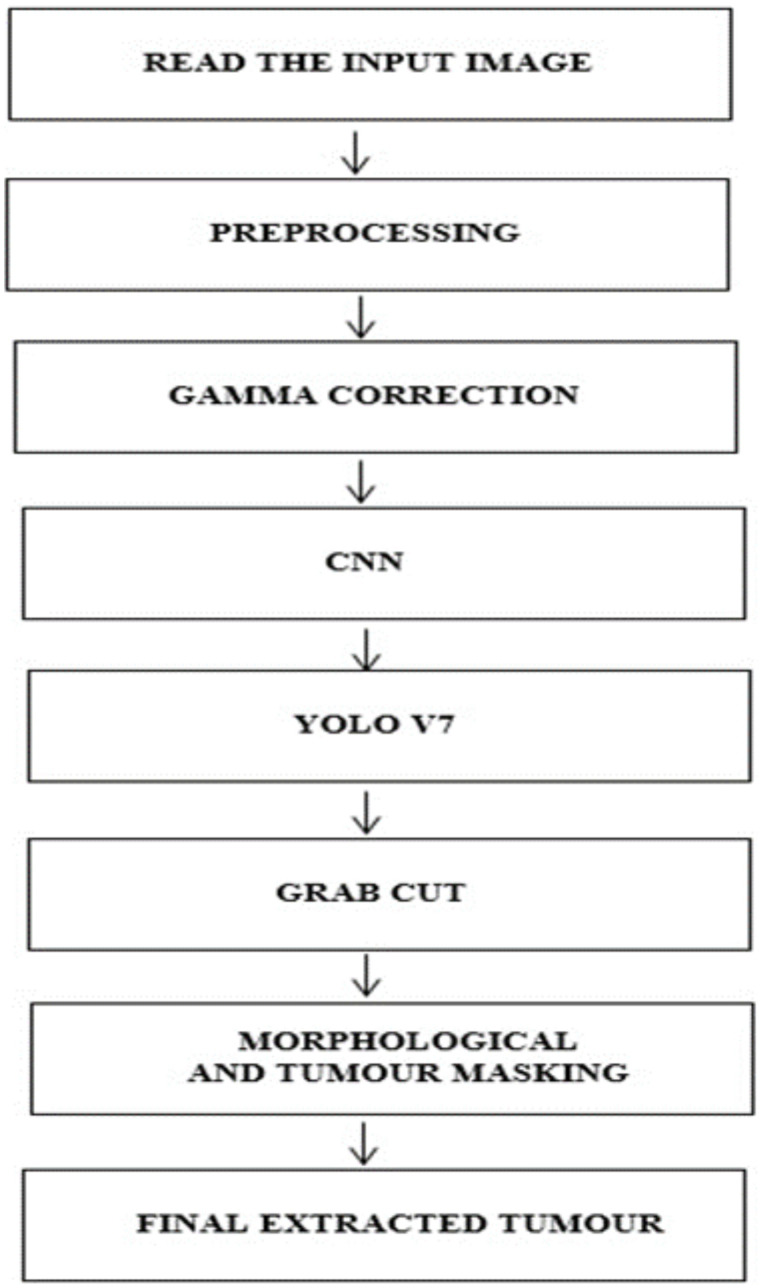
Workflow of the proposed method.

This work implements a structured approach to classify BTs. In the initial stage, the data from the brain tumor dataset is collected for analysis, and in the second stage, the images are preprocessed and subjected to Gamma correction in the third stage. In the fourth step, the YOLOv7 model is implemented to detect and locate the tumor. In the fifth stage, the Grab cut algorithm (37) is used for extracting the foreground of the tumor image The process flow of the proposed approach is shown in **Figure 1**.

### 2.1 Dataset collection and preparation

The dataset used for training the YOLOv7 model is collected from BR35H: Brain Tumor Detection 2020 (BR35H) (38). The dataset incorporated 1437 brain MRI images of which 734 were malignant and 703 were normal tumors. The dataset consists of both T1 and T2 weighted images and all images are two-dimensional (2D images) and have a dimension of 256 × 256 pixels. All the images are skull-stripped and labeled as ‘YES’ if the tumor is present; otherwise, labeled as ‘NO’. The description of the dataset is given in **Table 1**.

**Table 1:** Description of the Brain Tumor MRI Dataset.

	No of Images	No of patients	Training Samples	Testing Samples	Validation Samples	Label
With Tumor	734	68	514	110	110	Yes (1)
No Tumor	703	70	493	105	105	No (0)

The dataset is split into a ratio of 70:15:15 where 70% of the data is used for training the model, 15% for testing, and the remaining 15% for validation.

### 2.2 Data preprocessing

The data is preprocessed to enhance the quality of the images and make them suitable for the classification process. Preprocessing significantly improves the classification performance of the DL models by filtering out the uncertainties. In this work, preprocessing is performed using different stages such as RGB to Gray conversion, Otsu’s thresholding, Brain Skull Removal, Image Resizing, and Median filtering.

- **RGB to Gray conversion:** The RGB images consist of red, green, and blue-scale images arranged on top of each other. A grayscale image is a single-layered image denoted as an M × N array, whose values are used to represent the intensity of an image. To convert the RGB images into gray images, the components of the red, green, and blue images were extracted and represented in three different two-dimensional matrices. A new matrix is created with similar dimensions, where the number of rows and columns is equal to that of the RGB images. Each pixel of the RGB image was converted at location (i, j) to grayscale values by determining the weighted sum of the RGB color components and assigning it to the respective location (i, j) in the new matrix. This process simplifies the classification process and reduces computational complexity.
- **Otsu’s thresholding process:** This process is used to determine a threshold value to reduce the overlap between foreground and background images. In other words, Otsu’s algorithm returns a single intensity threshold value that separates the pixels into two different sets: foreground and background.
- **Brain Skull Removal:** Skull stripping was performed to eliminate non-brain tissue from the MRI images. This improved the speed and accuracy of the segmentation process. At this stage, flood-filling and masking operations were considered for the skull removal process. The flood-fill algorithm is used to identify or modify adjacent values in the image based on their similarity to the original values. Furthermore, masking was performed to identify a specific Region of Interest (ROI) for analyzing the tumor. This process discards image regions that are not characterized by tumors.
- **Image Resizing:** It is performed to minimize the size of the image without altering the actual image information. In this work, all the input images were resized to (250x250) pixels to avoid overfitting.
- **Noise Removal:** The noise in the input images was removed using a median filter with a kernel size of (3x3). Median filters are highly effective in filtering noise while preserving the edges. The filter computes the intensity of the pixel surrounded by the central pixel. The obtained median value was replaced with the intensity of the center pixel.

### 2.3 Gamma correction

The preprocessed images are subjected to Gamma correction to control the overall brightness of the tumor image. In this process, the images that are too dark or bright are corrected. The CNN performs automatic classification of the image attributes considering the statistical features. The contrast of the image is enhanced by dynamically modifying the parameters. In this stage, the Gamma correction is applied for each ROI of the image and this contributes to the overall image enhancement process. Overall, by incorporating gamma correction into the pipeline, the model can benefit from improved image quality, enhanced feature representation, and better generalization capabilities, ultimately leading to improved classification performance for brain tumor images. However, this did not change the underlying content or category of the image.

### 2.4 YOLOv7 model for tumor detection

Considering the benefits of the supervised learning of DL based YOLO model, this research employs an advanced version of the traditional YOLO model known as the YOLOv7 model. The YOLOv7 model is designed to develop an appropriate technique for identifying BT from brain MRI images. The working operation of YOLOv7 is unique and indistinct from fundamental methods used for detecting BT. In this process, the model simultaneously predicts the class and puts a bounding box around the tumor area. Each bounding box consists of five components (x, y, w, h, and the confidence score) with the first four components corresponding to the center coordinates (x, y, width, and height) of the respective bounding box, and the fundamental motive of YOLO is object detection and localization via bounding boxes. Therefore, two sets of bounding box vectors are required, i.e., vector ‘y’ is the representative of ground truth, and vector ‘Y’ is the predicted vector which is shown in Equation 1.


(1) $$Y\;=\left[p_{c},b_{x},b_{y},b_{h},b_{w},c\right]$$

- *p_c_* corresponds to the objectness score (the probability score of the grid containing an object).
- *b_x_*, *b_y_*, are the x and y coordinates of the center of the bounding box for the enveloping grid cell.
- *b_h_*, *b_w_*, correspond to the height and the width of the bounding box for the enveloping grid cell.
- ‘c’ corresponds to the class.

The MRI images are arranged in a grid of dimensions D x D for each grid cell. In the case where the center of the object of interest falls into one of the grid cells, that particular grid cell would be responsible for the detection of that object. This permitted the other cells to neglect the object in the case of multiple appearances. Each grid cell predicts B bounding boxes along with the dimensions and confidence scores. The confidence score was indicative of the absence or presence of an object within the bounding box. Therefore, the confidence score can be expressed as Equation 2:


(2) $$C=P_{r}\;\left(Object\right)\times IOU_{pred}^{truth}$$

where $P_{r}\;\left(Object\right)$ dignified the probability of the object being present, within a range of 0–1, with 0 indicating that the object does not exist and $\;IOU_{pred}^{truth}\;$ notes the intersection-over-union with the predicted bounding box for the ground truth bounding box. To address multiple bounding boxes containing no object or the same object, YOLO opts for non-maximum suppression (NMS). By defining a threshold value for the NMS, all overlapping predicted bounding boxes with an IoU lower than the defined NMS value are eliminated.

The losses associated with YOLOv7 are bounding box loss and objectness loss. Bounding box Loss (Localization loss) is represented in Equation 3:


(3) $${\text{L}}_{i\text{ box}}={\left(x_{i}-\hat{xi}\right)}^{2}+{\left(\text{yi}-\hat{y\text{i}}\right)}^{2}+{\left(\sqrt{\text{wi}}-\sqrt{\hat{\text{wi}}}\right)}^{2}+{\left(\sqrt{\text{hi}}-\sqrt{\hat{\text{hi}}}\right)}^{2}$$

here $\left(\hat{x_{i}},\hat{y_{i}},\hat{w_{i}},\hat{h_{i}}\right)$ represent ground truth values and $\;(x_{i}$ $\;y_{i}$ $\;w_{i,}\;h_{i}$ represent predicted values. Objectness Loss(confidence loss) is expressed as in Equation 4:


(4) $${\text{L}}_{\text{i}}\text{ object }=\left(c_{i}-\hat{c_{i}}\right)2$$

**Figure 2:**
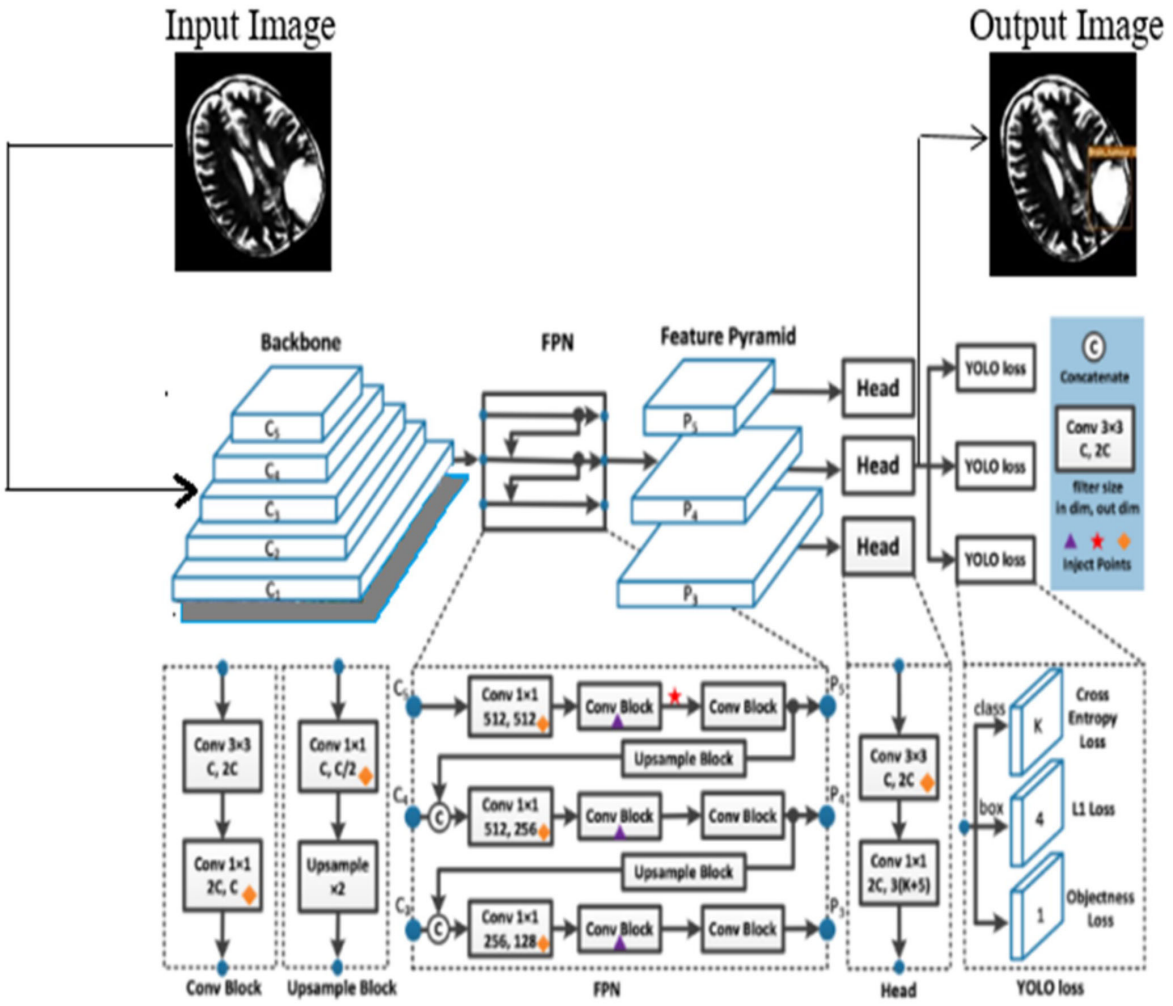
The proposed architecture of the YOLOv7 model.

In this process, the features are learned from labeled data, and the YOLOv7 is initialized using the learned features. In this work, the model is trained using both low-level and high-level features of the brain tumor, and the model is updated after every iteration. This allows fine-tuning of the learned parameters and enables the layers of YOLOv7 to capture features that are highly discriminative in nature. The architecture of the YOLOv7 model is illustrated in **Figure 2**.

The proposed YOLOv7 architecture incorporates three layers namely; (i) Backbone: E-ELAN, (ii) Neck: PANet, and (iii) Head: YOLO Layer. The backbone layer is the first layer responsible for extricating important tumor features from MRI images. A cross-stage partial network is utilized for extracting representational features.

**Figure 3:**
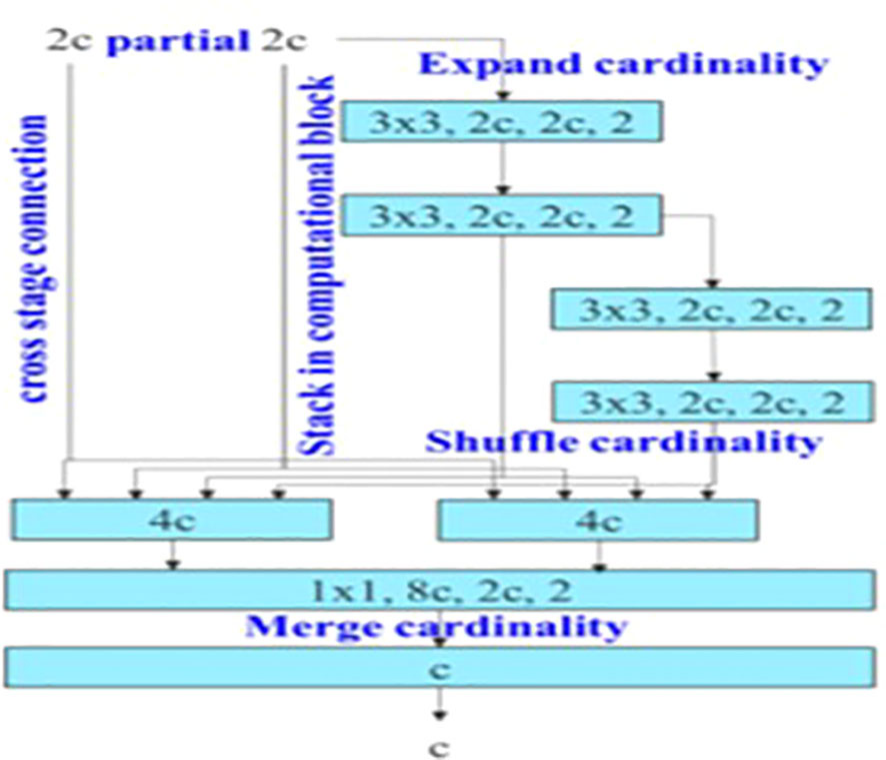
E-ELAN as Backbone Layer for YOLOv7 architecture.

The backbone of YOLOv7 consists of an Extended Efficient Layer Aggregation Network (E-ELAN) architecture (39) that uses expand, shuffle, and merge cardinality to improve the learning ability of the model without affecting gradient flow paths. E-ELAN modifies the YOLOv7 architecture in the computational block and the architecture remains the same in the transition layer. E-ELAN incorporates a group convolution method to maximize the channel capacity and cardinality of the computation block. The channel multiplier is applied to all blocks in the computation layer, and a feature map is created for each block. The feature maps from all blocks are concatenated, and the obtained feature map is used to merge the cardinality, as shown in **Figure 3**.

**Figure 4:**
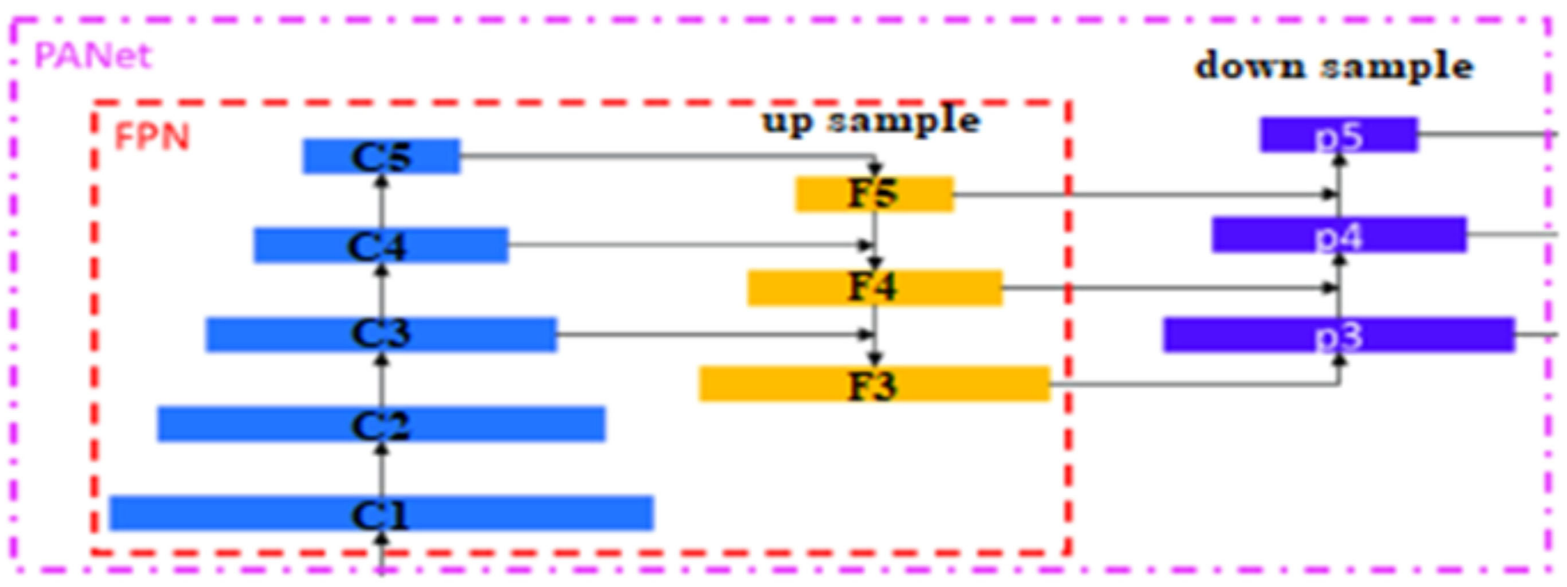
PANet layer in the YOLOv7 architecture.

The second layer is the PANet layer, also known as the neck of the model. The main reason behind selecting PANet is its capacity to restore the spatial data and thereby contribute significantly to the improvement of the localization process which in turn helps in creating the mask around the image. This layer employs anchor boxes for constructing feature vectors with bounding boxes for tumor detection. The neck aggregates the feature maps obtained from the Backbone and creates feature pyramids. The neck is made up of multiple paths and the features extracted from the backbone model are used to create the FPN as shown in **Figure 4**.

**Figure 5:**
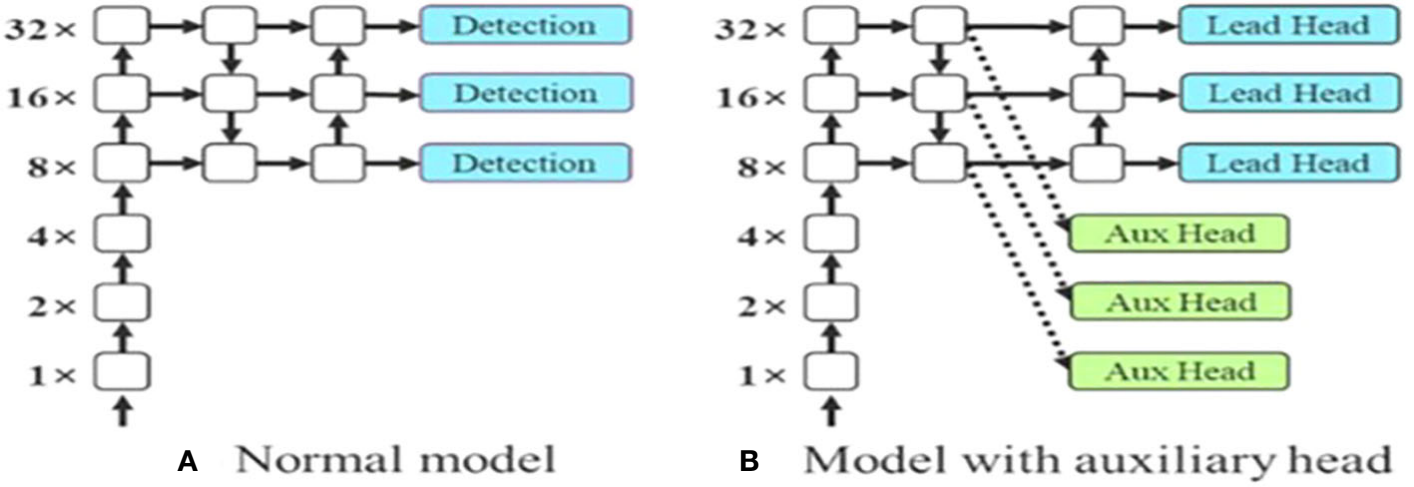
Head layer in YOLOv7 architecture. **(A)** Normal model **(B)** Model with auxiliary head.

The last layer in the YOLOv7 architecture is the head of the model which computes the final predictions as classification and localization. The head predicts classes and bounding boxes, classification scores, and objectness scores of objects based on the features collected from the neck. In YOLOv7, the head generates the final output, which is called the Lead Head, and assists in training the middle layers, called the Auxiliary Head. With the help of assistant loss, the weights of the auxiliary heads are updated, which enables deep supervision and thereby allows the model to learn better. The head of the YOLOv7 model is presented in **Figure 5**.

### 2.5 Grab cut algorithm

The detected tumor from the YOLOv7 model is analyzed using the Grab Cut algorithm which extracts the feature from the gamma-corrected image. This algorithm is used to extract the foreground of an image by drawing a rectangular box around it. This box helps in coordinating the image regions. However, the image contains both foreground and background regions and hence it is essential to eliminate the redundant background regions. This is achieved through a segmentation process wherein the pixels located in the foreground and background images are segmented and thereby helping in extracting only foreground images to achieve a better tumor detection performance. An input image is accepted whose value is 1 and for that, a bounding box is assigned. This determines the object in an image that needs to be segmented. The steps involved in the process are defined as follows:

Step 1: A Gaussian Mixture Model (GMM) is used for estimating the color distribution across the foreground and background images.

Step 2: A Markov random field is constructed over the pixel’s labels.

Step 3: The final segmented output images are obtained via the Grab Cut algorithm.

In Grab Cut, the model for monochrome images is replaced by GMM for color images. Soft segmentation is performed wherein a new vector k = {k_1_,…, k_n_,…, k_N_} is imputed to each pixel of GMM’s k_n_th component, where k_n_ = 1, 2, …, K (Normally K = 5), and α_n_ = 0,1 is assigned to each pixel to show that it belongs to either the foreground or background GMM. The energy function of the GrabCut algorithm is defined as shown in Equation 5:


(5) E (α, k, θ, z) = U (α, k, θ, z) + V (α, z)

and GMM is defined using Equation 6:


(6) $$G\left(z\right)=\;\sum_{i=1}^{K}\omega_{k\;}g_{k}\;\left(z;\;u_{k},\text{Σk}\right),\;\sum_{i=1}^{K}\omega_{k\;}=1,\;and\;0<\;\omega_{k\;}<1$$

Where g_k_ = (z; μ_k_, Σ_k_) is the Gaussian distribution function for each component t k, k = 1, 2,…K is given by Equation 7.


(7) $$ɡ\left(\;z;\;\mu ;\;\text{Σ}\right)=\frac{1}{\sqrt{2\pi^{D}\left|\sum \right|}}\exp[-\frac{1}{2}\;\left(z-\mu {)}^{T}\;{\sum^{}}^{-1}\left(Z-\;\mu \right)\right]$$

and ω_k_ is the weighting coefficient, μ_k_ is the means, Σ_k_ is the covariance matrix for k^th^ component and D is the number of dimensions of variable z. Combining equations 2, 3, and 4, the term U is computed as in Equation 8.


(8) $$U\;\left(\alpha ,\;k,\;\theta ,z\right)=\;\sum_{n}G\;\left(\alpha ,\;k,\;\theta ,z\right)$$

Where G (a, k, 0, z) is expressed as in Equation 9.


(9) $$\begin{array}{c} G\;\left(\alpha ,\;k,\;\theta ,z\right)=\;-log\omega \left(\alpha_{n},\;k_{n}\right)+\frac{1}{2}\text{log}|\sum \left(\alpha_{n},\;k_{n}\right)|\\ +\frac{1}{2}\;[z_{n}-\mu {\left(\alpha_{n},\;k_{n}\right)}^{T}\;\sum {\left(\alpha_{n},\;k_{n}\right)}^{-1}\left[z_{n}-\mu \left(\alpha_{n},\;k_{n}\right)\right]\; \end{array}$$

And the term θ is defined as in Equation 10.


(10) θ={π(α, k), μ (α, k), ∑(α, k), α=0, 1, k=1, …., K}

Grab Cut minimizes the energy function by modifying the iterative minimization cut algorithm. In the initial stage, the algorithm considers two-pixel sets wherein one set represents the background (αn = 0) and another one for object classes (α_n_ = 1). Two GMMs were initialized along with the two sets to start the iteration. GrabCut is an interactive version of graph cut where the user quickly marks some pixels as background, some as foreground, and then graph cut sorts out the rest (constraining the marked pixels to belong to the background and foreground or source-side and sink-side respectively). In this algorithm, the minimum cut is obtained by determining the maximum flow of data in the graph. In a graph, the connectivity is formed by removing the set of edges which also forms two individual subsets namely a maximum and a minimum cut. The max-flow min-cut theorem states that the maximum flow through any network from a given source to a given sink is equal to the minimum sum of a cut. The results of the simulation analysis are discussed in the below sections.

## 3 Results

This section provides localization and segmentation results on a dataset made publicly available on Kaggle (38). We conducted our experiments on the PYTHON 3.10.2 platform and executed on a system with an Intel(R) Core (TM) i5-1035G1 CPU, 8 GB RAM, and 3.3 GHz. We trained the model using the following hyperparameters: a learning rate (lr0) of 0.01, weight decay of 0.0005, and batch size of 16. We used the ADAM optimizer for 100 epochs.

### 3.1 Performance evaluation metrics

The efficacy of the YOLOv7 model was determined using the following metrics.

Accuracy is defined as the percentage of accurately detected brain tumors and is calculated as shown in Equation 11.


(11) $$\text{Accuracy}=\frac{TP+TN}{TP+TN+FP+FN\;\;}\;\;$$

Recall is defined as the ratio of brain tumor images that were accurately classified as shown in Equation 12.


(12) $$\text{Recall}=\frac{TP}{\;\;\;\;\;\;TP+FN\;\;\;\;\;\;\;\;\;\;}\;$$

The F1 score is determined as the weighted harmonic mean of its precision and recall are given by Equation 13.


(13) $$\text{F}1\text{ score}=\frac{2\;*\;Precision*Recall}{Precision\;+\;Recall}$$

Similarly, precision is defined as the accuracy of the positive predictions which is shown in Equation 14.


(14) $$\text{Precision}=\frac{TP}{TP+FP}$$

Based on the YOLOv7 model we trained, we achieved good results in terms of the overall mAP and individual class performance. The model achieved an overall mAP50 of 0.9391 and mAP 50-95 of 0.4981 on the validation set. This means that the model was able to accurately localize the tumor region with a high degree of confidence.

**Figure 6:**
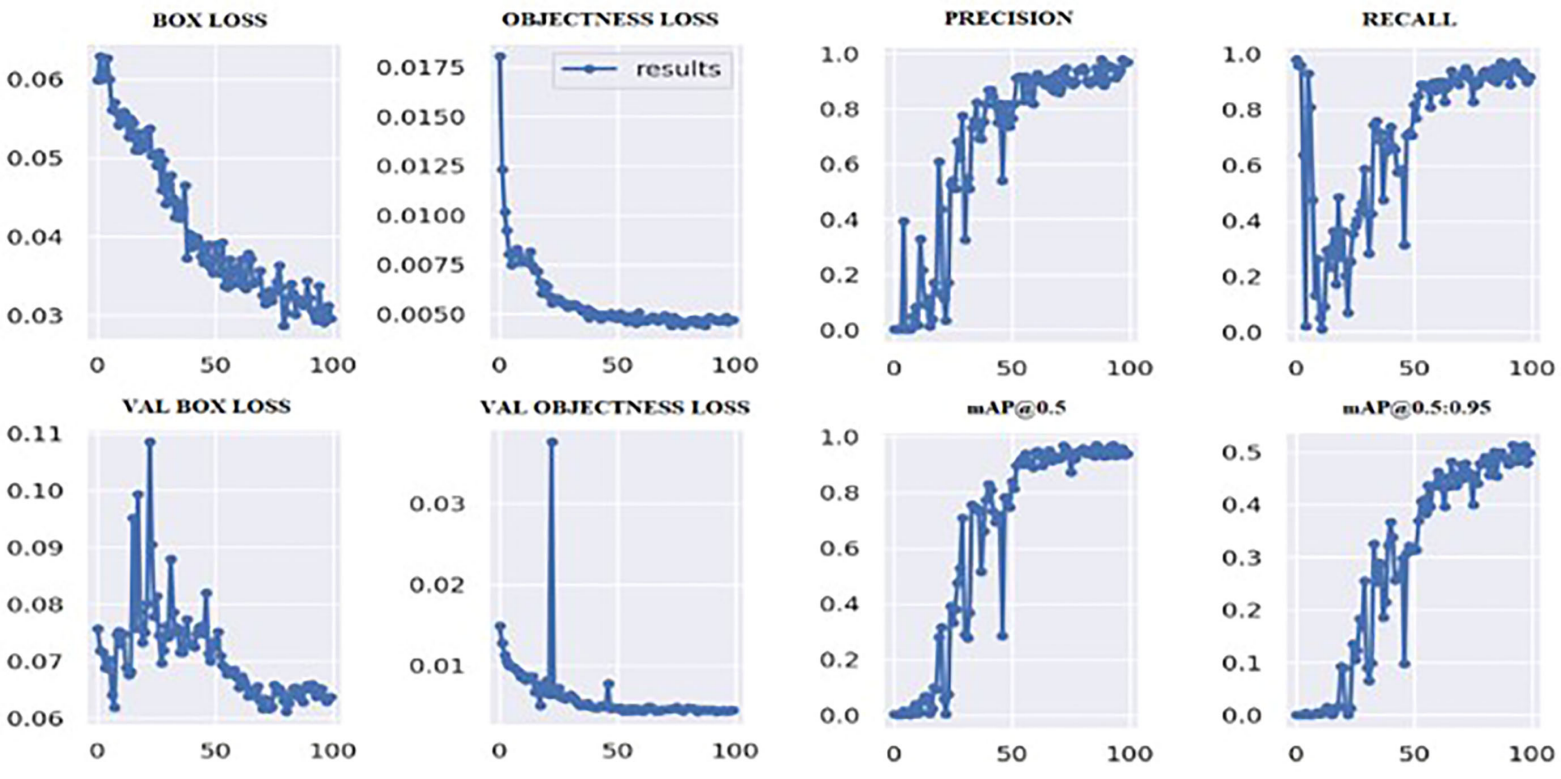
Outcomes of the training process.

**Figure 6** shows the loss values for the box loss, and object loss at each epoch during the training process. The box loss represents the difference between the predicted and ground-truth bounding box coordinates, and the object loss represents the confidence score for each object detected in an image. The goal of training an object detection model is to minimize the total loss, which is a combination of box loss, and object loss. The loss values should exhibit a decreasing trend as the training progresses, indicating an improvement in the model’s ability to localize the tumor region.

Moreover, from **Figure 6**, it appears that the precision, recall, and mean average precision (mAP) are all increasing with training epochs. This could indicate that the model improves over time and becomes more accurate at identifying the correct location of the tumor region. Our proposed model achieved a mean average precision (mAP50) of 0.9304 and 0.9391, respectively, indicating a high level of accuracy in identifying and localizing tumor regions in the images. The model accurately localized tumor regions with a precision (P) of 99% and recall (R) of 100%, demonstrating its ability to localize tumor regions even in challenging image conditions.

Overall, the results of our YOLOv7s model suggest that it performed well in accurately localizing the tumor region in the brain MR images we used for training and validation. Hence, we can infer that these results demonstrate the potential of the YOLOv7 and Grab cut model for localizing and extracting brain tumor in MR medical images.

### 3.2 Simulation results

**Figure 7:**
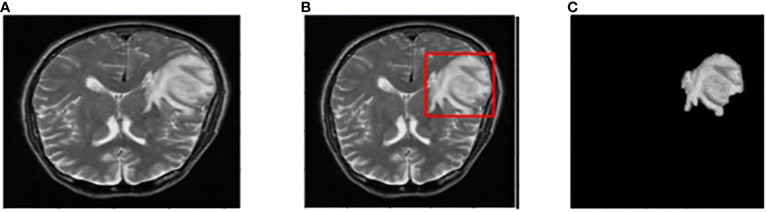
**(A)** Input image **(B)** Tumor detected using YOLOv7 **(C)** Extracted tumor using Grab Cut algorithm.

The input image for YOLOv7 and the tumor detected image is shown in **Figure 7**.

The values of different performance metrics obtained from simulation for the proposed method are tabulated in **Table 2**.

**Table 2:** Performance metrics for the proposed method.

	Training	Testing	Validation
**Accuracy**	99.5%	99.5%	99%
**Precision**	99.0%	99.0%	98.03%
**Recall**	100%	100%	100%
**Specificity**	100%	100%	100%
**F1 score**	99.5%	99.5%	99%

It can be inferred from the table that the proposed detection model achieved an optimal accuracy of 99.5% for training and testing datasets, and 99% for validation datasets. In addition to the performance evaluation metrics listed in **Table 2**, the performance of the proposed approach was validated in terms of training and validation loss, objectness loss, precision, and recall which are illustrated in the figures below.

**Figure 8:**
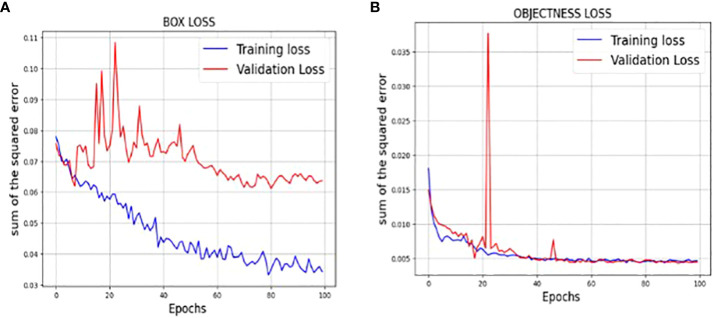
**(A)** Training and validation Box loss of the proposed model **(B)** Training and validation Objectness loss of the proposed model.

**Figure 9:**
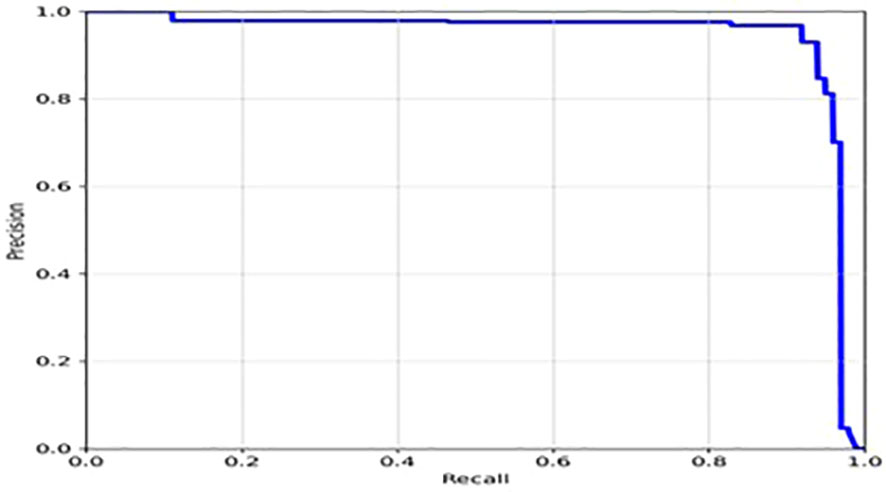
Precision- Recall of the proposed model.

The loss function of YOLOv7 is computed as a combination of two individual loss functions, that is, Bounding Box Regression (which measures how well predicted bounding boxes capture ground truth bounding boxes) and cross-entropy loss (which measures how well a job the detector did in predicting the correct class). The box loss represented in **Figure 8A** shows the effectiveness of the algorithm in terms of locating the center of the object (tumor image) and how well the predicted bounding box covers an object. The validation objectness loss is shown in **Figure 8B**. Objectness loss measures the probability that a tumor exists in the proposed ROI. If objectness is high, the image window is likely to contain an object. As observed in **Figure 8B**, the proposed approach exhibits a high objectness score and hence helps in locating the tumor from the given image. The precision and recall graphs for the proposed model are shown in **Figure 9**.

The training measurement values used to train the YOLOv7 model are tabulated in **Table 3**. A graphical representation of the training process outcomes is shown in **Figure 6**.

**Table 3:** Findings from training procedure.

Epochs	Box loss	Val Box loss	Objectness loss	Val Objectness loss	Precision	Recall	mAP @ 0.5	mAP@ 0.5: 0.95
1	0.07792	0.07574	0.01807	0.01491	0.00332	0.9798	0.0032	0.00048
10	0.06179	0.07527	0.00763	0.00861	0.08104	0.2626	0.03995	0.00600
20	0.05863	0.07332	0.00650	0.00729	0.6102	0.2688	0.2806	0.09291
30	0.04947	0.07618	0.00532	0.00612	0.7755	0.5859	0.7094	0.2555
40	0.0455	0.07286	0.05112	0.00469	0.8157	0.7152	0.773	0.323
50	0.03831	0.06884	0.00461	0.00460	0.9157	0.8889	0.9211	0.4099
60	0.04114	0.06723	0.00506	0.00475	0.8183	0.8687	0.8892	0.4378
70	0.04045	0.06302	0.00479	0.00465	0.9261	0.9091	0.9291	0.4515
80	0.03322	0.06304	0.00456	0.00465	0.9384	0.9293	0.9464	0.4771
90	0.03923	0.06588	0.00482	0.00449	0.8846	0.9291	0.9304	0.4881
100	0.03426	0.06369	0.00469	0.00449	0.9681	0.9191	0.9391	0.4981

**Figure 6** shows that the mAP values obtained during the validation for 100 epochs was IOU = 0.5 and mAP for IOU from 0.5 to 0.95. For mAP at 0.5 and 0.95 are measured as the step values for different values such as 0.05 (0.5, 0.55, 0.6, 0.65, 0.7, 0.75, 0.8, 0.85, 0.9, 0.95).

**Figure 10:**
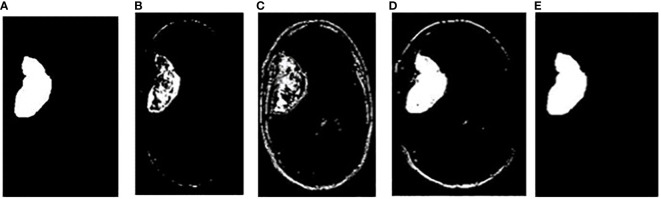
**(A)** Ground truth image **(B)** Fuzzy segmented image **(C)** K-means segmented image **(D)** Otsu’s segmented image **(E)** Proposed Grab Cut segmented image.

In addition, the comparison is done between ground truth test images and predicted test images. In this work, four segmentation techniques like Fuzzy C means segmentation, K-means clustering, Otsu thresholding, and Grab cut algorithm are applied for finding the DICE similarity measurement between ground truth images and predicted test images. The resultant images for the segmentation process are shown in **Figure 10**.

**Figure 11:**
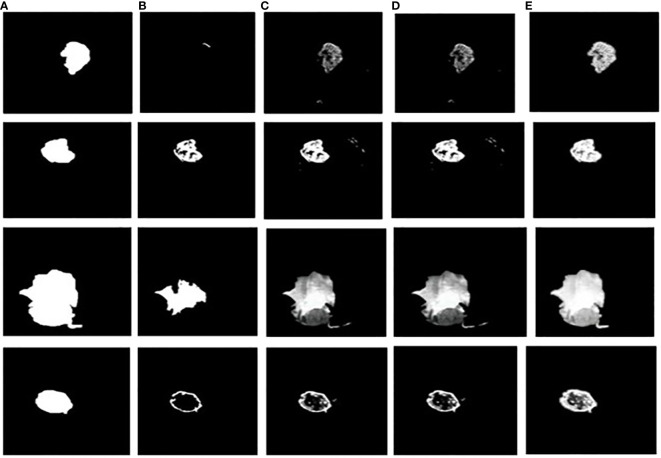
Tumor extracted images **(A)** Ground truth image **(B)** CNN-SVM + Grab cut **(C)** YOLOv5 + Grab cut **(D)** YOLOv6 + Grab cut **(E)** YOLOv7 + Grab cut.

The dice similarity measurements for all four segmentation techniques are illustrated in **Table 4**. It is understood from the results that the proposed Grab cut algorithm yields enhanced results compared to other techniques by achieving high dice similarity measurement. The performance of the YOLOv7 model in combination with the Grab cut algorithm is also compared with that of other tumor extraction mechanisms, and the results are illustrated in **Figure 11**.

**Table 4:** Dice similarity measurement of various segmentation techniques.

Segmentation techniques	Dice similarity measurements
Fuzzy Segmentation(Pitchai, R et al.,2021)	0.9240
K-means Clustering(Sinaga, K. P et al.,2020)	0.9354
Otsu’s Thresholding(Huang, C et al., 2021)	0.8765
**Proposed Grab cut Algorithm**	**0.9831**

Bold text and values represent the proposed work.

## 4 Discussion

Although several studies have been conducted on the application of deep learning for tumor localization and extraction, the combination of Grab cut and YOLOv7 has not been widely employed in this field. Actually, as far as we are aware, no studies have used YOLOv7 plus Grab cut for this purpose. Therefore, by using YOLOv7 in combination with Grab cut for tumor localization and extraction, our work represents a novel contribution to the field.

Moreover, it is important to remember that accuracy on its own might not be a good enough statistic for object detection tasks because it ignores false positives and false negatives. Rather, mean average precision, or mAP, is frequently employed to assess how well object detection models perform. The mAP offers a more thorough assessment of the model’s performance by accounting for precision and recall at various intersection over union (IoU) thresholds. Our study’s mAP50 of 0.9391 shows that our model does a good job of identifying the tumor location.

**Figure 12:**
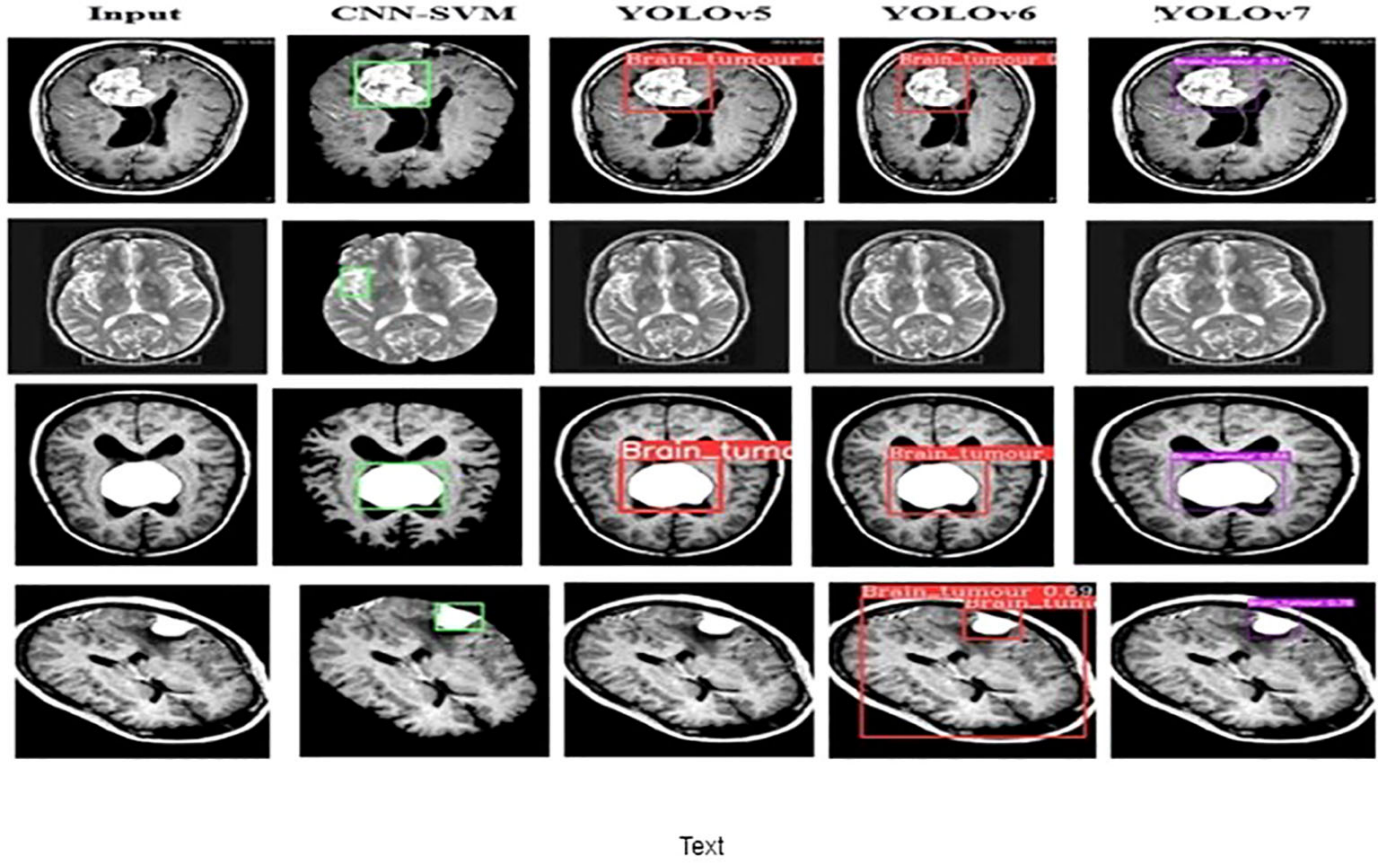
Brain Tumor detected images of the different models..

The performance of the proposed model is compared with other techniques, such as the hybrid CNN-SVM, YOLOv5, and YOLOv6 models. The outcomes are shown in **Figure 12**, and the obtained values are listed in **Table 5**. The table clearly shows that our model outperforms the benchmark models in terms of metrics of dice similarity, accuracy, precision, recall, specificity, and F1 score.

**Table 5:** Comparison of the proposed method with other methods.

	Hybrid CNN-SVM	YOLOv5	YOLOv6	Proposed YOLOv7
**Accuracy**	69%	97.5%	97.5%	**99%**
**Precision**	69.79%	97.02%	97.79%	**98.03%**
**Recall**	67%	98%	97%	**100%**
**Specificity**	67%	98%	97%	**100%**
**F1 score**	68.36%	97.51%	97.48%	**99%**

Bold text and values represent the proposed work.

In **Figure 10**, the first, third, and fourth rows represent the tumor input image and the second row represents a non-tumor input image. As inferred from the comparative results (**Table 4**) the proposed YOLOv7 achieves excellent results compared to the existing methodologies. The accuracy of 99% is obtained by using the proposed approach and the accuracy of hybrid CNN-SVM is 69%, YOLOv5 and YOLOv6 are 97.5% respectively. A highest precision of 98.03% is achieved by the YOLOv7 model and the precision values are 69.79%, 97.02% and 97.79% for hybrid CNN-SVM, YOLOv5 and YOLOv6 techniques respectively.

**Figure 13:**
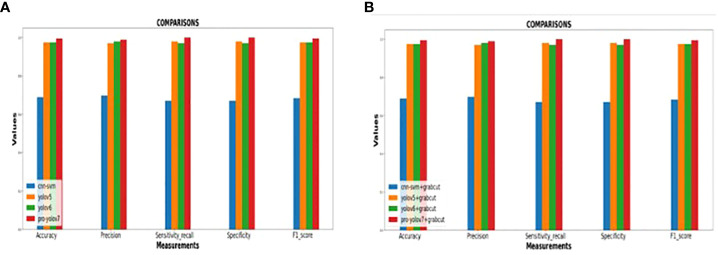
**(A)** Performance of the various models without Grab cut algorithm **(B)**Performance of the various models with Grab cut algorithm.

**Figure 14:**
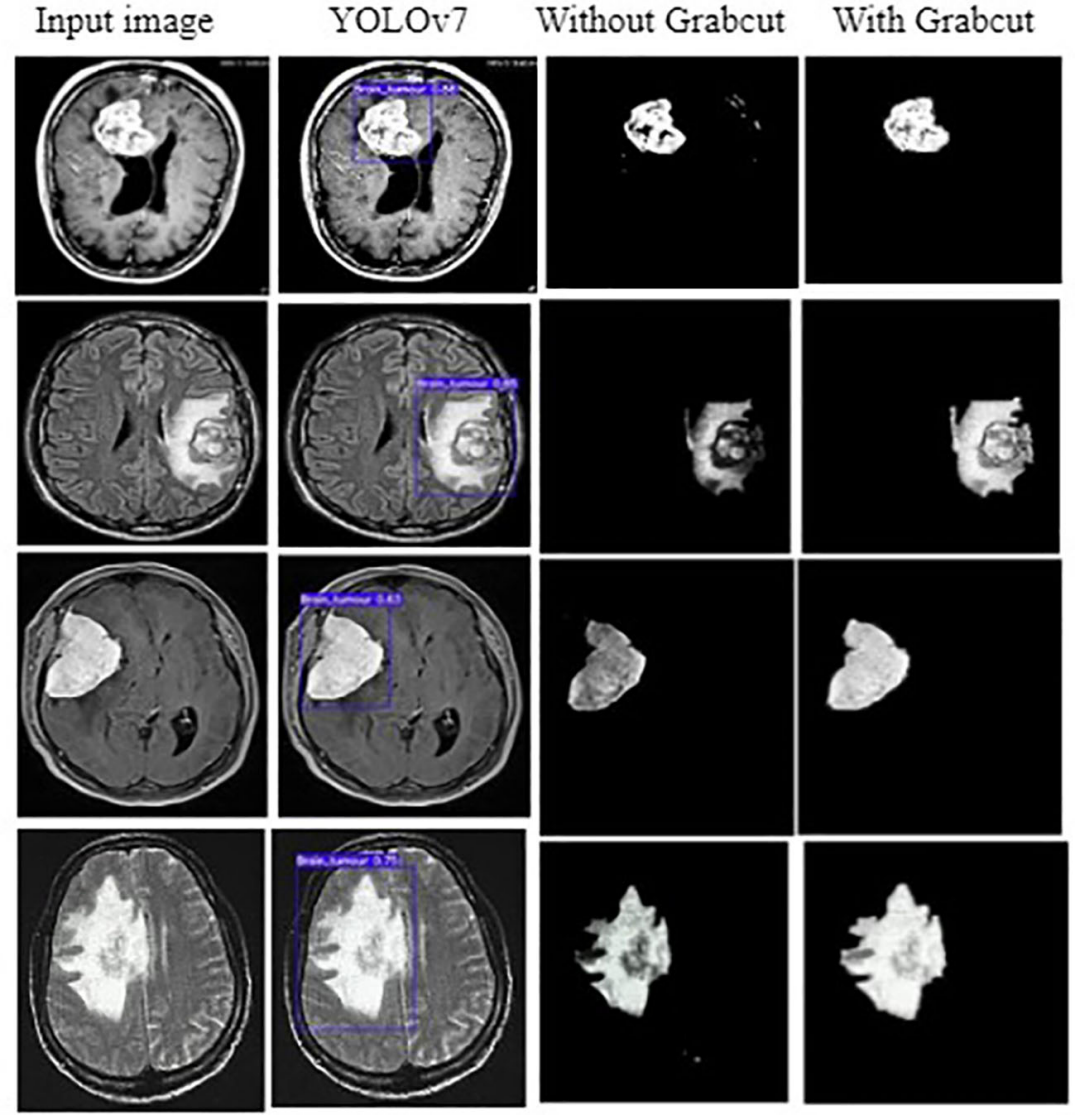
Outcomes of YOLOv7 with and without Grabcut.

In addition, the YOLOv7 model is also tested with and without the application of the Grab cut algorithm, as shown in **Figures 13A, B**, respectively. The average mean dice similarity score value between the predicted test images and corresponding ground truth images using Grab cut algorithm for tumor extraction is shown in **Table 6**. The outcomes of YOLOv7 with and without the combination of Grab Cut are shown in **Figure 14**.

**Table 6:** Dice similarity measurement of various models with Grab Cut algorithm.

	Hybrid CNN-SVM + Grab Cut	YOLOv5 + Grab Cut	Yolov6 + Grab Cut	Proposed Yolov7 + Grab Cut
Dice Similarity Measurements	0.3328	0.8105	0.8190	0.9147

Results show that the proposed technique attains a high dice score between tumor extracted images and Ground truth images. The findings show that the performance of the YOLOv7 model is improved by the inclusion of the Grab Cut algorithm as compared to the performance of the model without the algorithm.

## 5 Conclusion

This paper deploys a new BT detection and extraction method using deep convolution neural network YOLOv7 in combination with Grab cut algorithm. This approach detects the salient images for accurate results. The proposed model involved different stages of preprocessing like noise removal, image resizing, thresholding and RGB to gray conversion. The tumor image is converted to grayscale before being segmented and corrected using the Gamma correction process based on the threshold level. Our methodology provides better resolution and dimension-independent segmentation outcomes than the prior deep learning-based detection techniques. We evaluated our method using BR35H: Brain Tumor Detection 2020 (BR35H) dataset. Results show that the YOLOv7 model in combination with grab cut achieves an outstanding accuracy of 99% in comparison to existing hybrid CNN-SVM, YOLOv5 and YOLOv6 models. The outcome of the analysis reveals that the YOLOv7 model is fast compared to the other models. In addition, the YOLOv7 accurately detects and extracts BT in the presence of the Grab Cut algorithm. This approach is best identified for BT detection when implemented for larger datasets. This model can be extended in the future to explore various types of tumors from the extracted tumor for accurate diagnosis.

## Data availability statement

The original contributions presented in the study are included in the article/supplementary material. Further inquiries can be directed to the corresponding author.

## Author contributions

SK: Conceptualization, Formal analysis, Methodology, Writing – original draft. YK: Supervision, Validation, Writing – review & editing.
